# Histone Demethylase UTX is an Essential Factor for Zygotic Genome Activation and Regulates Zscan4 Expression in Mouse Embryos

**DOI:** 10.7150/ijbs.34635

**Published:** 2019-08-24

**Authors:** Lige Bai, Lei Yang, Caiquan Zhao, Lishuang Song, Xuefei Liu, Chunling Bai, Guanghua Su, Zhuying Wei, Guangpeng Li

**Affiliations:** 1 State Key Laboratory of Reproductive Regulation and Breeding of Grassland Livestock (R2BGL), Inner Mongolia University, Hohhot, China.; 2 College of Life Sciences, Inner Mongolia University, Hohhot, China.

**Keywords:** Embryo, Zygotic genome activation, *Utx*, * Zscan4d*, 2-cell retardation

## Abstract

Following fertilization, the zygotic genome is activated through a process termed zygotic genome activation (ZGA), which enables zygotic gene products to replace the maternal products and initiates early embryonic development. During the ZGA period, the embryonic epigenome experiences extensive recodifications. The H3K27me3 demethylase UTX is essential for post-implantation embryonic development. However, it remains unclear whether UTX participates in preimplantation development, especially during the ZGA process. In the present study, we showed that either knockdown or overexpression of UTX led to embryonic development retardation, whereas simultaneous depletion of UTX and overexpression of ZSCAN4D rescued the embryonic development, indicating that UTX positively regulated *Zscan4d* expression. Using a transgenic mice model, we also found that UTX was required for preimplantation embryonic development. In conclusion, these results indicate that UTX functions as a novel regulator and plays critical roles during ZGA in addition to early embryonic development.

## Introduction

Following fertilization, the totipotent zygote experiences a series of cleavage stages that eventually result in the formation of a blastocyst 1-4. During this period, the gametes transform into embryos in a process termed the maternal to zygotic transition (MZT). MZT involves the degradation of maternal products and the transcriptional activation of the zygotic genome 5-7. Zygotic genome activation (ZGA) is a process that coordinates the original quiescent zygotic genome to become transcriptionally active 8-10. During the onset of ZGA, a portion of genes are highly expressed; these are called the ZGA markers. The correct expression of ZGA markers is critical for embryonic development. Therefore, understanding the associated molecular mechanisms of ZGA will facilitate the improvement of embryonic development rates consequent to nuclear transfer and assisted reproductive technologies.

Ubiquitously transcribed X chromosome tetratricopeptide repeat protein (UTX, also known as KDM6A) function as a histone demethylase and is responsible for removing histone tags at trimethylated lysine 27 of histone H3 (H3K27me3) 11, 12. UTX mutations have been discovered in patients with Kabuki syndrome, a rare genetic disorder (1/32,000 births) characterized by distinct facial features, intellectual disability, short stature and dermatoglyphic and skeletal abnormalities 13, 14. UTX regulates gene activation by mediating a functional interaction between the lineage-defining T-box transcription factor family and a Brg1-containing SWI/SNF remodeling complex 15. In addition, UTX has been shown to be pivotal for several developmental and biological processes including embryogenesis, cardiac development, muscular development, somatic cell reprogramming, aging, and cancer 12, 16-20. Furthermore, UTX is essential for the resolution and activation of numerous retinoic acid-inducible bivalent genes during the RA-driven differentiation of mouse embryonic stem cells (ESCs) 21, 22. However, the effect of UTX activation during the ZGA process is still not well understood.

The zinc finger and SCAN domain containing 4 (*Zscan4*) gene cluster contains 6 transcribed paralogous genes (*Zscan4a-f*) that have high sequence similarities and are thus collectively called *Zscan4* 23. *Zscan4d* is specifically expressed in 2-cell stage embryos, whereas *Zscan4c* is transcribed predominantly in ESCs 24. Both *Zscan4c* and* Zscan4d* have four DNA binding zinc-finger domains and a SCAN domain, which is predicted to mediate protein-protein interactions 25. In ESCs, knockdown of *Zscan4* shortens the telomeres, increases karyotype abnormalities and spontaneous sister chromatid exchanges, and retards cell proliferation 25. Furthermore, knockdown of *Zscan4d* in mouse zygotes disrupts the ZGA process, impairs embryonic development, and causes 2-cell retardation 23.

In this study, we demonstrated that the histone demethylase UTX is critical for preimplantation embryonic development. Combined with the *in vitro* (knockdown &. overexpression) and *in vivo* (transgenic mice) experiments, we found that either knockdown or overexpression of *Utx* induced 2-cell embryo retardation and reduced embryonic development. In addition, the expression of *Zscan4d* was significantly dysregulated in the *in vitro* and *in vivo* experimental groups. Taken together, these results demonstrated that UTX is an essential factor for ZGA and regulates *Zscan4* expression in mouse embryos. Here, we proposed a novel insight regarding the function of UTX during the ZGA process in early embryonic development.

## Results

### Knockdown of UTX leads to 2-cell retardation

**Figure 1:**
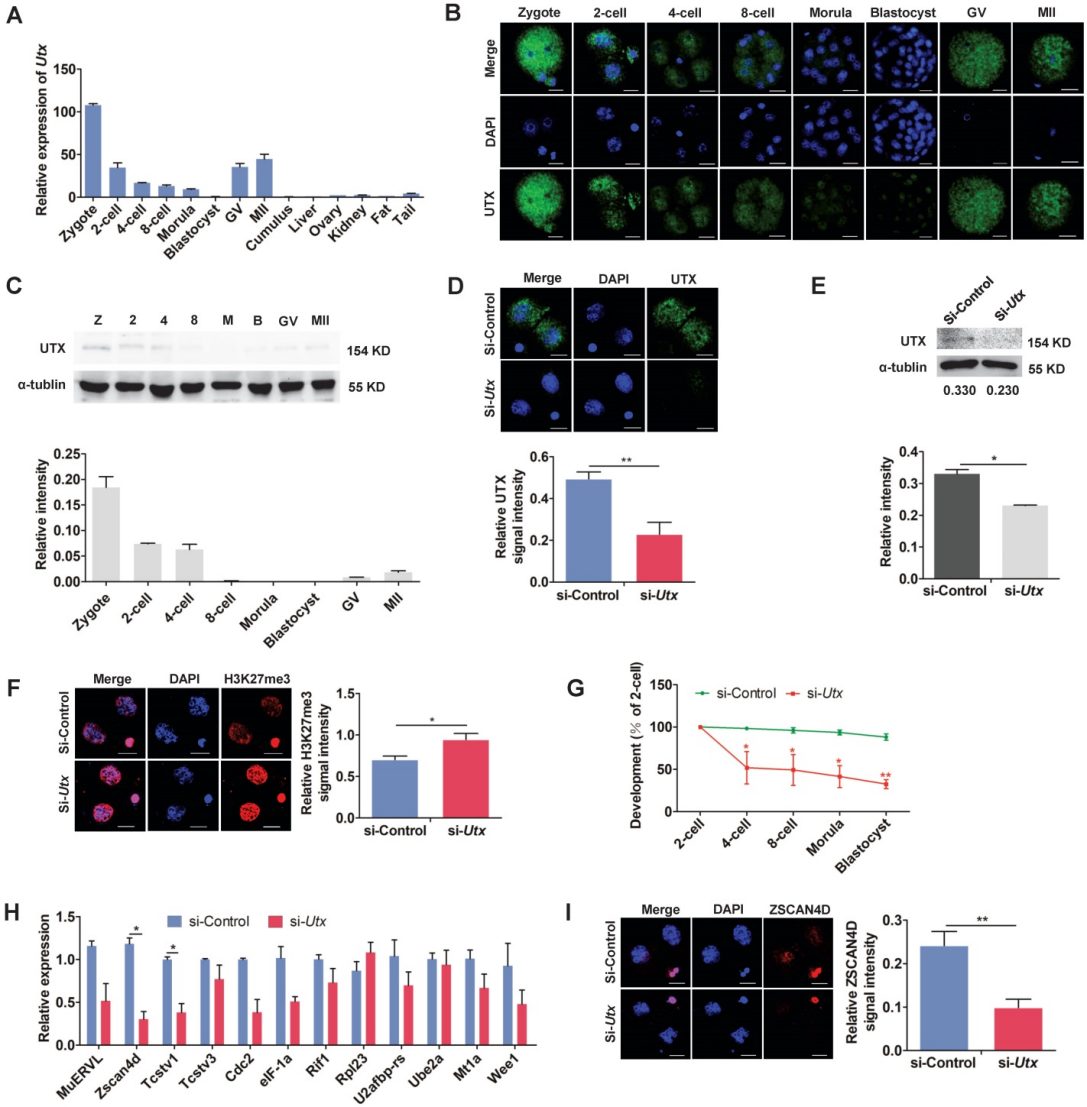
**Knockdown of UTX leads to 2-cell developmental retardation.** (A) qPCR results showing the mRNA levels of *Utx* in mouse embryos and tissues. Error bars indicate SEM. All values were normalized to *Gapdh*, *n* = 3. (B) Immunofluorescence staining using an anti-UTX antibody at the zygote, 2-cell, 4-cell, 8-cell, morula, blastocyst, GV, and MII oocyte stages, respectively. Representative images from ≥ 20 embryos analyzed in four independent micromanipulations for each condition are shown. Scale bar, 20 μm. (C) Western blot analysis of UTX levels at the zygote, 2-cell, 4-cell, 8-cell, morula, blastocyst, GV, and MII oocyte stages (upper), and the quantification of UTX intensity (bottom). Representative images reflect one of three independent experiments; protein lysates from 500 embryos were loaded in each lane, normalized to total α-tubulin, and measured using ImageJ software. Z, zygote; 2, 2-cell; 4, 4-cell; 8, 8-cell; M, morula; B, blastocyst; GV, GV oocyte; MII, MII oocyte. Error bars indicate SEM. *n* = 3. (D) Representative immunofluorescence images of UTX expression in the 2-cell stage embryos injected with *Utx*-siRNA (upper), and the quantification of UTX signal intensity (bottom). Representative images from ≥ 20 embryos analyzed in four independent micromanipulations for each condition are shown. Scale bar, 20 μm. For immunofluorescence quantification, bar graphs show the relative intensities of UTX/DAPI signal ratios. Error bars indicate SEM. **P < 0.01 by the two‐tailed Student's *t*‐test. (E) Western blot analysis of UTX levels in the 2-cell stage embryos injected with *Utx*-siRNA (upper), and quantification of UTX intensity (bottom). Representative images reflect one of three independent experiments; protein lysates from 500 embryos were loaded in each lane; numbers below the western blot indicate band intensity, normalized to total α-tubulin, and measured using ImageJ software. Error bars indicate SEM. *n* = 3. *P < 0.05 by the two-tailed Student's *t*-test. (F) Representative immunofluorescence images of H3K27me3 expression in the 2-cell stage embryos injected with *Utx*-siRNA (left), and the quantification of H3K27me3 signal intensity (right). Representative images from ≥ 20 embryos analyzed in four independent micromanipulations for each condition are shown. Scale bar, 20 μm. For immunofluorescence quantification, bar graphs show the relative intensities of H3K27me3/DAPI signal ratios. Error bars indicate SEM. *P < 0.05 by the two‐tailed Student's *t*‐test. (G) Embryonic development rate of si-*Utx*-injected embryos cultured *in vitro*. The efficiency was calculated based on the number of 2‐cell embryos. Error bars indicate SD, *n* ≥ 3. *P < 0.05, **P < 0.01 by the two-tailed Student's *t*-test. (H) qPCR results showing mRNA levels of ZGA markers in si-*Utx*-injected 2-cell embryos. Error bars indicate SEM. All values were normalized to *Gapdh*. *n* = 3. *P < 0.05 by the two-tailed Student's *t*-test. (I) Immunofluorescence of ZSCAN4D in si-*Utx*-injected embryos at the 2-cell stage (left); quantification of ZSCAN4D signal intensity (right). For the immunofluorescence images, bar graphs show the relative intensities of ZSCAN4D/DAPI signal ratios. Error bars indicate SEM. Representative images from ≥ 20 embryos analyzed in Image J; independent micromanipulations for each condition are shown. Scale bar, 20 μm. **P < 0.01 by the two-tailed Student's *t*-test. Scale bar, 20 μm.

In order to understand the pattern of UTX expression, we collected oocytes, embryos, and different tissues. The qPCR, IF and WB results showed that UTX expression was predominant in the preimplantation embryos, especially in the zygotes and 2-cell stage embryos (Figure 1A, 1B, and 1C).

To explore the role of UTX in early embryonic development, we designed and constructed two short interfering RNAs (siRNAs) specifically targeting *Utx*. The two siRNAs were mixed in a 1:1 ratio to form *Utx*-siRNA (Figure S1A). A siRNA without any specificity to* Utx* or other genes was constructed as a si-control. The results of qPCR analyses revealed that the *Utx* mRNA expression level decreased by > 90% in the si-*Utx*-injected embryos compared to that in the si-control-injected embryos (*P* < 0.01; Figure S1B, S1C). Consistent with the qPCR results, the expression of UTX protein was significantly decreased in the si-*Utx*-injected embryos as shown using IF and WB (IF, *P* < 0.01; WB, *P* < 0.05; Figure 1D, 1E). Furthermore, IF staining showed that the H3K27me3 levels in the si-*Utx*-injected group were significantly increased (*P* < 0.05; Figure 1F). Notably, the embryonic development rate from the 4-cell to blastocyst stage was significantly reduced in si-*Utx*-injected group (Figure 1G). Based on these results, we hypothesized that knockdown of *Utx* will influenced the occurrence of the ZGA event.

To confirm this hypothesis, we selected 12 ZGA markers and 4 maternal effector genes to detect their expression in 2-cell stage embryos by qPCR. Compared with the si-control group, the expression of *Zscan4d* and *Tcstv1* was significantly down-regulated, whereas the other ZGA-associated genes were not affected (*P* < 0.05; Figure 1H). The expression of the maternal effector gene *Stella* was significantly lower than that in the si-control group (*P* < 0.01; Figure S1D). In addition, IF results showed that the expression of ZSCAN4D protein in the si-*Utx-*injected group was significantly down-regulated (*P* < 0.01; Figure 1I). These results indicated that knockdown of UTX impaired embryonic development and resulted in abnormal expression of ZSCAN4D at both mRNA and protein levels.

### Overexpression of UTX also results in 2-cell retardation

**Figure 2:**
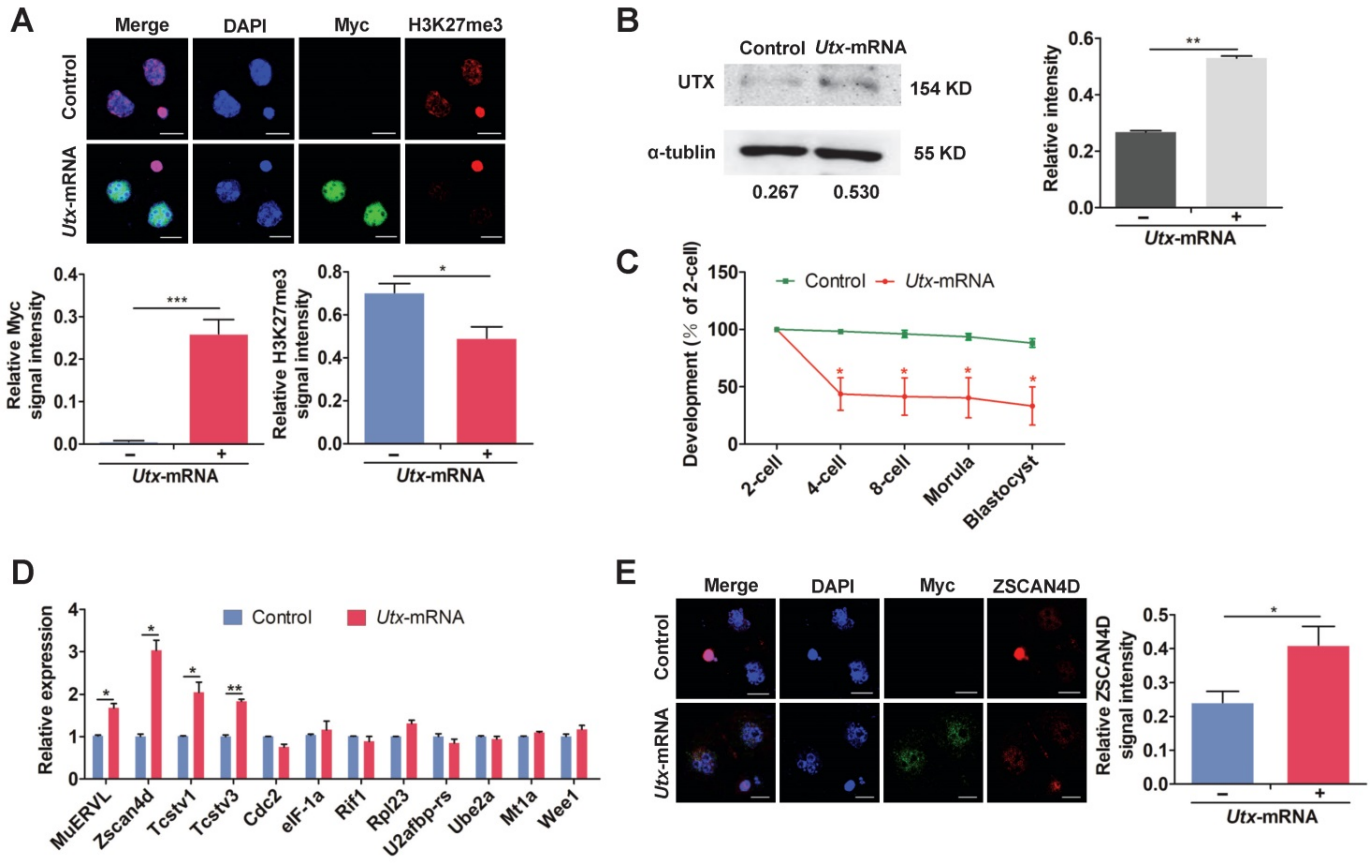
**Overexpression of UTX results in 2-cell developmental retardation.** (A) Representative immunofluorescence of Myc and H3K27me3 expression in 2-cell embryos injected with *Utx*-mRNA (upper), and quantification of Myc and H3K27me3 signal intensities (bottom). Representative images from ≥ 20 embryos analyzed in Image J; independent micromanipulations for each condition are shown. For quantification, bar graphs show the relative intensities of Myc/DAPI or H3K27me3/DAPI signal ratios. Error bars indicate SEM. *P < 0.05, ***P < 0.001 by the two-tailed Student's *t*-test. Scale bar, 20 μm. (B) Western blot analysis of UTX levels in 2-cell embryos injected with *Utx*-mRNA (left), and quantification of UTX intensity (right). Representative images reflect one of three independent experiments; protein lysates from 500 embryos were loaded in each lane; numbers below the western blot indicate band intensity, normalized to total α-tubulin, and measured using ImageJ software. Error bars indicate SEM. *n* = 3. **P < 0.01 by the two-tailed Student's *t*-test. (C) Embryonic development rate of *Utx*-mRNA-injected embryos cultured *in vitro*. The efficiency was calculated based on the number of 2-cell embryos. Error bars indicate SD, *n* ≥ 3. *P < 0.05 by the two-tailed Student's *t*-test. (D) qPCR results showing mRNA levels of ZGA markers in *Utx*-mRNA-injected 2-cell embryos. Error bars indicate SEM. All values were normalized to *Gapdh*. *P < 0.05, **P < 0.01 by the two-tailed Student's *t*-test. (E) Immunofluorescence of Myc and ZSCAN4D in *Utx*-mRNA-injected embryos at the 2-cell stage (left), and quantification of ZSCAN4D signal intensity (right). For the immunofluorescence images, bar graphs show the relative intensities of ZSCAN4D/DAPI signal ratios. Error bars indicate SEM. Representative images from ≥ 20 embryos analyzed using Image J; independent micromanipulations for each condition are shown. Scale bar, 20 μm. *P < 0.05 by the two-tailed Student's *t*-test.

To determine the effects of UTX overexpression, we constructed an *in vitro* transcription vector containing *Utx* N-terminally tagged with Myc tag (Figure S2A, S2B). It is worth noting that the exogenous Myc ectopic expression vector allowed us to track the UTX protein in early embryos without the use of specific antibodies. The qPCR assay showed that *Utx* mRNA expression in *Utx*-mRNA-injected group was nearly 6-fold higher than that in the control group at the 2-cell stage (*P* < 0.01; Figure S2C, S2D). Consistent with the qPCR results, the expression of UTX protein was significantly increased in the *Utx*-mRNA-injected group as shown using IF and WB (IF,* P* < 0.001; WB, *P* < 0.01; Figure 2A, 2B). In addition, IF staining showed that overexpression of UTX markedly reduced the levels of H3K27me3 (*P* < 0.05; Figure 2A).

The majority of *Utx*-mRNA-injected embryos arrested at the 2-cell stage, whereas the control group developed to the blastocyst stage (Figure 2C). This phenomenon was consistent with results from the si-*Utx*-injected group. Compared with the control group, a portion of ZGA-associated genes including *MuERVL*, *Zscan4d, Tcstv1*, and *Tcstv3* were significantly highly expressed in the *Utx*-mRNA-injected embryos, as detected by qPCR (*MuERVL*, *Zscan4d*, *Tcstv1*, *P* < 0.05; *Tcstv3*,* P* < 0.01; Figure 2D). The expression of *Lin28a*, one of the four maternal effector genes, was significantly up-regulated in the *Utx* mRNA-injected embryos (*P* < 0.05; Figure S2E). Furthermore, the expression of ZSCAN4D protein was significantly up-regulated in the *Utx*-mRNA-injected embryos as shown using IF assay, which was consistent with the qPCR result (*P* < 0.05, Figure 2E). Taken together, these results suggested that overexpression of UTX also impaired embryonic development and led to abnormal expression of ZSCAN4D.

### *Utx* transgenic mice exhibit ZGA defects

**Figure 3:**
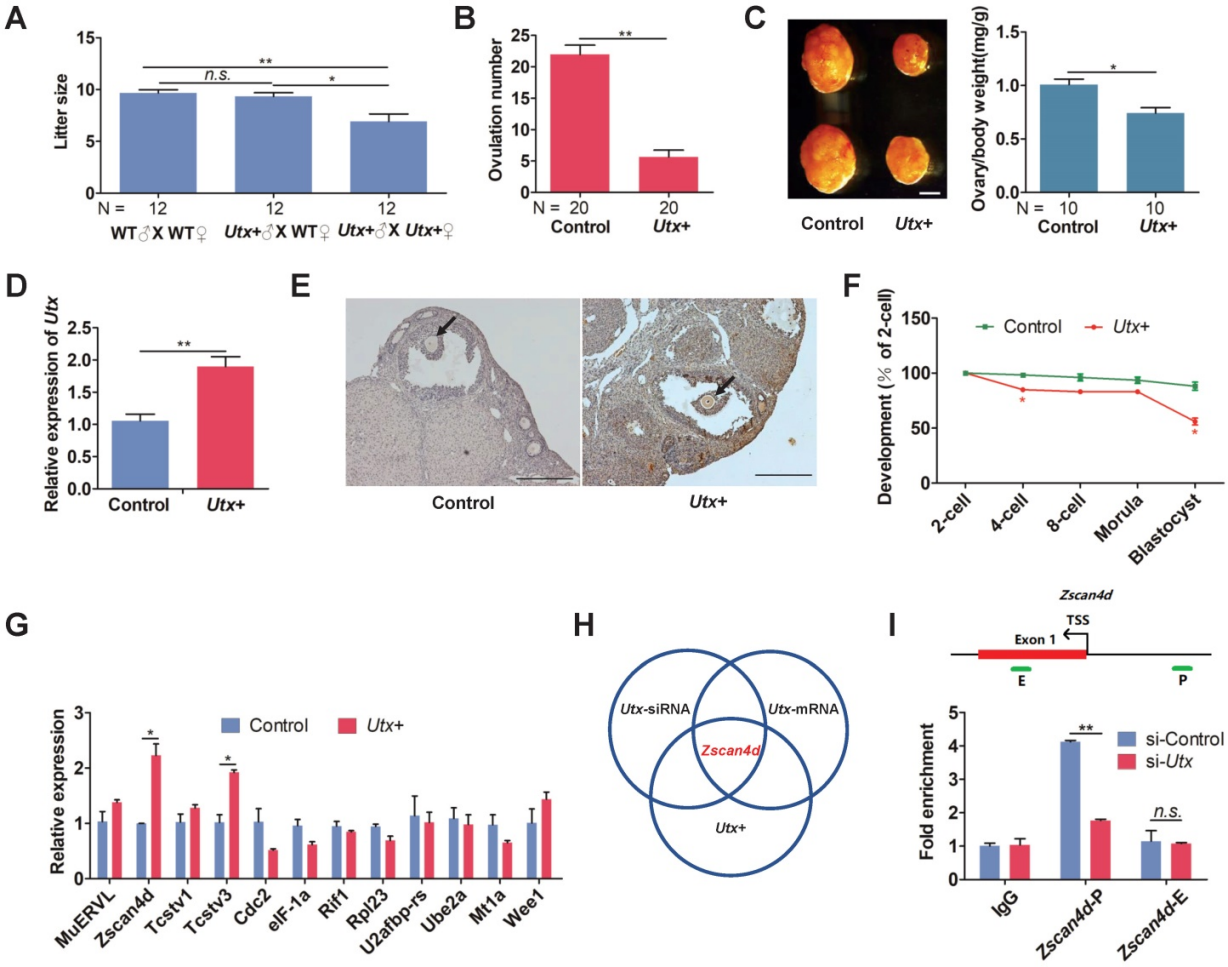
***Utx* transgenic mice also exhibit ZGA defects.** (A) Numbers of pups per litter. ♂, ♀, and X indicate the male, female, and cross mate, respectively. WT, wild-type; N, total number of couples; *n.s.*, not significant. Error bars indicate SEM. *P < 0.05, **P < 0.01 by the two-tailed Student's *t*-test. (B) Ovulation number of *Utx*+ females following hCG injection. N, total number of females. Error bars indicate SEM. **P < 0.01 by the two-tailed Student's *t*-test. (C) Images representative of the control and *Utx*+ mice ovary (left), bar graphs showing the ratio of ovary/body weight (right). N, total number of females. Error bars indicate SEM. *P < 0.05 by the two-tailed Student's *t*-test. Scale bar, 500 μm. (D) qPCR results showing mRNA levels of *Utx* in *Utx*+ mice 2-cell embryos. Error bars indicate SEM. Values were normalized to *Gapdh*. **P < 0.01 by the two-tailed Student's *t*-test. (E) Myc immunohistochemistry staining in wild-type (WT) and *Utx*+ mice on ovaries prepared from 8-week-old mice. Black arrows indicate oocytes. Scale bar, 200 μm. (F) Developmental rates in the *Utx*+ mice embryos cultured *in vitro*. The efficiency was calculated based on the number of 2-cell embryos. Error bars indicate SD, *n* ≥ 3. *P < 0.05 by the two-tailed Student's *t*-test. (G) qPCR results showing mRNA levels of ZGA markers in *Utx*+ mice 2-cell embryos. Error bars indicate SEM. All values were normalized to *Gapdh*. *P < 0.05 by the two-tailed Student's *t*-test. (H) Schematic illustration of the target gene of UTX. (I) Location of PCR amplicons in the *Zscan4d* gene used for ChIP analyses (upper). ChIP-qPCR assays with an anti-UTX antibody at the indicated *Zscan4d* locus in si-control and si-*Utx*-injected 2-cell embryos (*Zscan4d*-E: exon 1 region,* Zscan4d*-P: promoter region) (bottom). Error bars indicate SEM. IgG served as the control. **P < 0.01 by the two-tailed Student's *t*-test. E, Exon 1; P, promoter; TSS, transcription start site;* n.s.*, not significant

Having demonstrated that both knockdown and overexpression of UTX markedly reduced embryonic development rate, we further generated *Utx* transgenic mice as an *in vivo* model (referred to as *Utx*+; Figure S3A, S3B) to explore whether* Utx*+ mice had similar phenomenon to the* in vitro* experiment. The body weight of the *Utx*+ mice showed that neither male nor female mice presented any significant difference compared to that controls, respectively (Figure S3C). When *Utx*+ males and females were inter-crossed, the number of newborn pups was significantly lower than that from ♂ (*Utx*+) X ♀ (wild-type) or wild-type inter-crossed groups (Figure 3A). This result indicated that the overexpression of UTX could reduce the fecundity of females. In addition, the number of ovulations in *Utx*+ females were significantly reduced (*P* < 0.01; Figure 3B). Furthermore, the size of their ovaries was significantly smaller than that of the controls, and the ovary/body weight ratio also being significantly decreased (*P* < 0.05; Figure 3C). As expected, the *Utx* gene significantly highly expressed at the 2-cell embryos as shown by qPCR (*P* < 0.01; Figure 3D). In the ovaries, the UTX proteins highly expressed in oocytes as shown by immunohistochemistry (IHC) (Figure 3E).

The blastocyst formation rate of the *Utx*+ embryos was significantly reduced, which was in consistence with the *Utx*-mRNA-injected embryos (Figure 3F). The qPCR results demonstrated that expression of the ZGA-associated genes *Zscan4d* and *Tcstv3* was significantly up-regulated (*P* < 0.05; Figure 3G), as was expression of the maternal effector gene* Gdf9* (*P* < 0.05; Figure S3D). These findings suggested that the pattern of gene expression in the *Utx*+ mice was identical to that in the *Utx*-mRNA-injected group. Together, the results from *Utx*-siRNA, *Utx*-mRNA, and the *in vivo Utx*+ mice model indicated that *Zscan4d* represents the target gene of UTX (Figure 3H). To test whether UTX positively regulated *Zscan4d* in 2-cell embryos, we performed a ChIP-qPCR experiment using an anti-UTX antibody and two specific primer pairs for the promoter (P) and exon 1 (E) of the *Zscan4d* locus. The results confirmed that UTX does bind to the *Zscan4d* promoter region (Figure 3I). Furthermore, the UTX occupancy at *Zscan4d*-P region declined following si-*Utx* injection (*P* < 0.01; Figure 3I), supporting the concept that *Zscan4d* expression can be positively regulated by UTX.

### Fine-tuning *Zscan4d* expression partially rescue si-*Utx* embryonic development

To further examine the relationship between UTX and Zscan4d, we constructed an *in vitro* transcription vector wherein *Zscan4d* was tagged N-terminally with Myc tag (Figure S4A, S4B). The qPCR and IF results showed that the expression of ZSCAN4D was significantly increased in the *Zscan4d*-mRNA-injected group compared to that in control group (qPCR, *P* < 0.01; IF, *P* < 0.001; Figure S4C, S4D).

**Figure 4:**
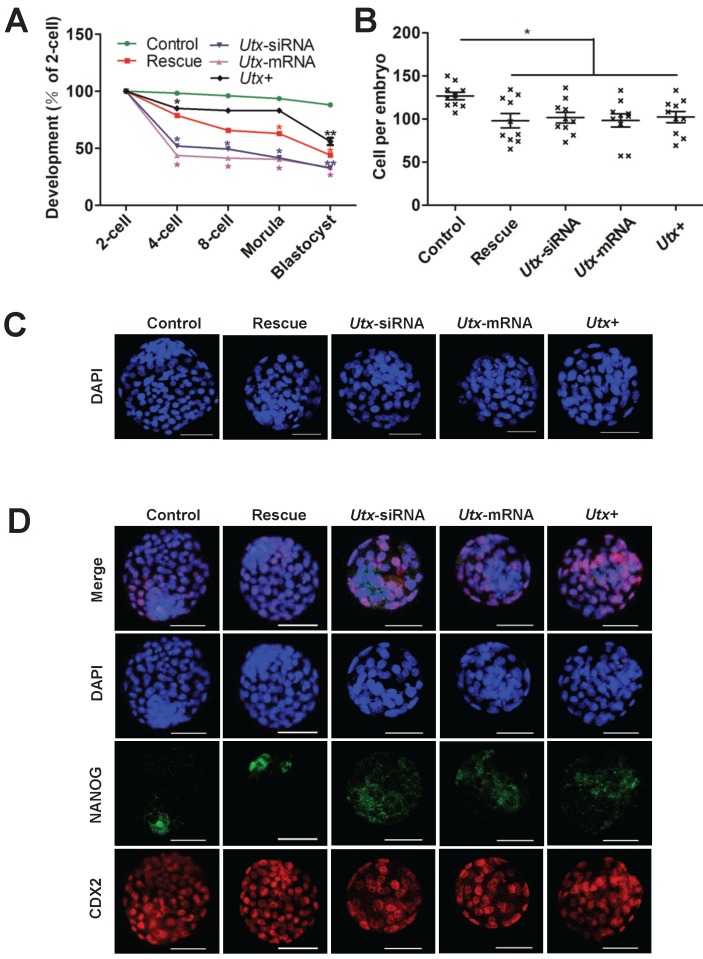
**Fine-tuning expression of *Zscan4d* partially rescues si-*Utx* embryonic development.** (A) Developmental rates in the rescued (*Utx*-siRNA+*Zscan4d*-mRNA) and other groups (Control, *Utx*-siRNA, *Utx*-mRNA, *Utx*+) of mouse embryos cultured *in vitro*. The efficiency was calculated based on the number of 2-cell embryos. Error bars indicate SD, *n* ≥ 3. *P < 0.05, ** P < 0.01 by the two-tailed Student's *t*-test. (B) and (C) Cell numbers of blastocyst determined by the nuclei stained by DAPI and representative DAPI staining of blastocysts of the rescue (*Utx*-siRNA+*Zscan4d*-mRNA) and other groups (Control, *Utx*-siRNA, *Utx*-mRNA, *Utx*+) of mouse embryos after 120 h of culture *in vitro*. Error bars indicate SEM. *P < 0.05 by the two-tailed Student's *t*-test. Scale bar, 50 μm. (D) Immunofluorescence images of the rescue (*Utx*-siRNA+*Zscan4d*-mRNA) and other groups (*Utx*-siRNA, *Utx*-mRNA, *Utx*+) of mouse embryos after 120 h of culture *in vitro*. NANOG (ICM) and CDX2 (TE) were used as lineage markers. Representative images from ≥ 20 embryos analyzed in independent micromanipulations for each condition are shown. Scale bar, 50 μm.

To explore whether the abnormal embryonic development following UTX modulation is mainly caused by *Zscan4d* dysregulation, we performed the following rescued experiments in the *Utx*-knockdown embryos. The *Utx*-siRNA and *Zscan4d*-mRNA were simultaneously injected into the zygotes, which showed that the embryonic development significantly improved in the rescued group, especially from 2-cell to 4-cell stage (Figure 4A, Figure S5, Figure S6, and Table S1). However, the blastocyst formation rate did not significantly increase (Figure 4A, Figure S5, Figure S6, and Table S1), and the cell numbers of blastocysts was with no difference between different injected groups (Figure 4B, 4C). To gain further insights into blastocyst lineage segregation, the blastocysts derived from different group were subjected to IF staining of NANOG and CDX2 (Figure 4D). The results showed that the NANOG and CDX2 were exclusively localized to the nuclei of the inner cell mass (ICM) and trophectoderm (TE) in the control group, while the NANOG signals was mislocalized to the ICM in the *Utx*-siRNA, *Utx*-mRNA, and *Utx*+ groups (Figure 4D). These results indicated that fine-tuning expression of *Zscan4d* partially rescue the impaired embryonic development upon UTX depletion.

## Discussion

Previous reports have indicated that ZGA is important for embryonic development, as the embryos will be arrested at the 2-cell stage when ZGA is blocked 19, 26. Within a very short period, embryonic genomes undergo dramatic epigenetic remodeling from the maternal pattern to the reestablished zygotic pattern. During the ZGA process, the *MuERVL* repeats belonging to type III endogenous retroviruses are specifically expressed at the 2-cell stage 27 as is *Zscan4d* among the *Zscan4* gene cluster of zinc-finger proteins, which has been demonstrated to have a critical role in telomere elongation and genomic stability 25, 28. In addition, the H3K27me3 modification is considered to constitute an essential mechanism for the regulation of ZGA owing to its association with gene transcriptional repression 29. In turn, the UTX protein, a specific demethylase for H3K27me3, has recently emerged as a key regulator of many important biological events including regulation of the pluripotency of stem cells and induced pluripotent stem cells 30. UTX is also involved in embryonic axial patterning formation and controls the H3K27me3 modification level at the *Hox* gene cluster 31, 32.

The content of* Utx* mRNA is abundant in parthenogenetic embryos, whereas its transcripts begin to degrade following mitosis 12. MII oocytes have the unique ability to reprogram a somatic cell nucleus into a totipotent state via somatic cell nuclear transfer (SCNT). Our previous study indicated that excessive H3K27me3 modifications would lead to ZGA failure during SCNT reprogramming 19. Furthermore, we demonstrated that injection with *Utx* mRNA could facilitate the ZGA of cloned embryos and improve the developmental potential. We were able to efficiently obtain SCNT blastocysts via *Utx* mRNA injection but failed to obtain live cloned pups. In the present study, we observed ectopic expression of UTX in zygotes resulted in 2-cell retardation, which implied that successful ZGA is essential for the development of fertilized or SCNT-derived embryos. Nonetheless, fertilized embryos completely differ from SCNT-derived embryos. SCNT embryos are generated by directly injecting somatic nuclei into enucleated oocytes, whereas the oocyte cytoplasm is evolutionally designed to reprogram the spermatozoa. Therefore, although H3K27m3 functions as a SCNT reprogramming barrier, its effects on the reprogramming process likely differ between sperm and somatic cells. It is known that the occurrence of UTX defect affects the SCNT embryo development. However, it remains unknown whether UTX is actively involved in the process of fertilized embryo development. In the present study, results from both *in vitro* and *in vivo* indicated that either knockdown or overexpression of UTX induced 2-cell embryo retardation and reduced embryonic development. UTX positively targeted to the *Zscan4d* gene and directly induced changes in *Zscan4d* gene expression. Fine-tuning the expression of the *Zscan4d* gene partially rescued the development of si-*Utx*-injected embryos. Similarly, results from Falco et al. have shown that *Zscan4d* is essential for mouse embryonic development through the regulation of blastocyst formation 23. Together, these results suggested that UTX is essential for the ZGA and is targeted to the *Zscan4d* gene.

To further examine whether UTX is critical to *Zscan4d* expression, we simultaneously depleted *Utx* and overexpressed *Zscan4d*, and found that overexpression of *Zscan4d* in the *Utx*-knockdown embryos partially rescued embryonic development, especially from 2-cell to 4-cell stage. These results further supported the hypothesis that *Zscan4d* is a downstream gene of UTX. Recently, we and others independently demonstrated that the endogenous retrovirus gene *MuERVL* plays essential roles in ESCs and preimplantation embryos 19, 27, 33-36. Nevertheless, the functions and biochemical properties of the* MuERVL* gene have yet to be fully identified. A recent report also demonstrated that reduced H3K27me3 resulted in the up-regulation of ZGA marker genes in mouse embryos 37. These results suggested that the H3K27me3 demethylase UTX serves as a novel *MuERVL* regulator and is essential for preimplantation embryonic development. In addition, a global decrease in H3K27me3 occurs during embryonic cleavage stages of bovine and porcine development 38-40, which further implies that UTX plays critical roles during early embryonic development. Moreover, UTX regulates the ZGA probably be via recruitment of transcription activators or chromatin remodeling factors 15.

The present study highlights the previously unknown role for UTX in *Zscan4d* gene expression in the ZGA process. Both knockdown and overexpression of UTX induced retardation of 2-cell embryos, decreased the embryonic development, and affected expression of *Zscan4d*. Thus, the histone demethylase UTX influenced the occurrence of ZGA, which provide a novel insight into the function of UTX in ZGA process in early embryonic development. Future studies will be necessary to determine whether other UTX co-regulators might activate ZGA.

## Materials and Methods

### Ethics statement

All studies adhered to procedures are consistent with the National Research Council Guide for the Care and Use of Laboratory Animals and were approved by the Institutional Animal Care and Use Committee at Inner Mongolia University.

### Animals

B6D2F1 (C57BL/6 × DBA/2) female and male mice were purchased at 6-8 weeks of age from Inner Mongolia University (China). Pseudopregnant CD1 mice were used as embryo recipients. All mice were reared in house under specific-pathogen-free conditions and were housed under controlled lighting conditions (light: 08:00~20:00). The animals had free access to food and water. The mice were randomly allocated to each experimental group.

### Plasmids and antibodies

The inserts of plasmids pcs2-UTX and pcs2-ZSCAN4D used in this study were PCR-amplified from the cDNA of NIH-3T3 cells and 2-cell mouse embryos, respectively. The PCR products were cloned into the *Xho* I and *Xba* I (Thermo, USA) sites downstream of the Myc ectopic tag. The antibodies covered were rabbit anti-UTX (Millipore, USA, for IF and WB), rabbit anti-H3K27me3 (Abcam, USA, for IF), mouse anti-Myc (Santa Cruz, USA, for IF), rabbit anti-ZSCAN4 (Millipore, for IF), rabbit anti-CDX2 (Abcam, for IF), mouse anti-NANOG (Abcam, for IF), rabbit α-tubulin (Proteintech, USA, for WB), Alexa Fluor 594 and Alexa Fluor 488 (Thermo, for IF), anti-UTX (KDM6A) (Abcam, for ChIP), anti-IgG (Abcam, for ChIP).

### Transgenic mice generation

Transgenic mice were produced on a B6D2F1 background. For pronuclear microinjection, the pcs2-UTX expression vector was linearized using *Sal* I and *Not* I (Thermo). The purified DNA was diluted in 1×TE buffer (10 ng/μl) and microinjected into the well-recognized pronuclei of zygotes. The injected zygotes were transferred into the oviducts of pseudopregnant females. Transgenic mice were identified by PCR analysis using specific primers (Table S2).

### Collection of oocytes and fertilized embryos

Superovulation was performed as previously described 19. The GV stage oocytes were isolated from B6D2F1 female (6-8 weeks) mice 46 h following pregnant mare serum gonadotropin (PMSG, Sansheng, China) injection. The MII stage oocytes were isolated from B6D2F1 female (6-8 weeks) mice 16 h following human chorionic gonadotropin (hCG, Sansheng) injection. Zygotes (24 h after hCG) were obtained from the ampulla of the superovulated female mice after mating with male mice. Then, 2-cell (44 h after hCG), 4-cell (56 h after hCG), 8-cell (67 h after hCG), morula (76 h after hCG), and blastocyst (97 h after hCG) embryos were obtained by culturing zygotes in KSOM medium.

### *In vitro* mRNA transcription, siRNA construction, and microinjection in zygotes

*Utx* cDNAs and *Zscan4d* cDNAs were cloned into SP6-driven vectors. To prepare mRNAs for microinjection, *Utx* and *Zscan4d* expression vectors were linearized by *Sal* I and *Not* I (Thermo), the constructs were then purified with phenol-chloroform extraction and ethanol precipitation. The mRNA was synthesized by *in vitro* transcription using a mMESSAGE mMACHINE SP6 Ultra Kit (Thermo) according to the manufacturer's instructions. The synthesized mRNA was purified by lithium chloride precipitation and diluted with nuclease-free water. For mRNA injection, zygotes were injected with 100 ng/μl mRNA. The *Utx*-siRNA-1 and *Utx*-siRNA-2 respectively located at 585 and 1,518 bp downstream from the start codon (4390771- s75838/s75839, Thermo). The commercially available siRNA without any specificity to the known genes was used as control (4390843, Thermo). The siRNAs were diluted to 20 μM in nuclease-free water and stored at -80°C until use. As previously described 12 with minor modifications, the zygotes were transferred to the manipulation M2 media and the mRNA or siRNA were microinjected into the zygotes with approximately 10 pl volume using FemtoJet (Eppendorf, German) micromanipulator. Following injection, the zygotes were kept at room temperature for 15 min and then incubated in the incubator.

### RNA extraction and quantitative real-time PCR

Total RNA was extracted from each pool of embryos (n = 3 pools of 20 embryos) using the Pico-Pure RNA Isolation Kit (Thermo). The reverse transcription-qPCR (RT-qPCR) was transcribed from total RNA using the SuperScript III First-Strand Synthesis System (Thermo) according to the manufacturer's instructions. All primers were listed in Table S2. The quantitative real-time PCR (qPCR) was performed using a TB Green Premix Ex Taq (Takara, Japan), and the signals were detected with ABI7500 Real-Time PCR System (Thermo). Analyses of the relative gene expression was measured using the 2-ΔΔCt method.

### Immunofluorescence staining and immunohistochemistry

As previously described 12, the immunofluorescence (IF) staining of embryos conducted with minor modifications. The embryos were rinsed three times in phosphate-buffered saline (PBS) with 0.3% (m/v) bovine serum albumin (BSA), fixed with 4% paraformaldehyde for 1 h and then permeabilized with 0.5% Triton X-100 for 15 min at room temperature. The embryos were blocked with 3% (m/v) BSA in PBS for 1 h and then incubated with the primary antibodies for 1 h at 37°C. After washing three times with PBS containing 0.3% BSA, the embryos were incubated with the secondary antibody for 1 h at 37°C. The nuclei were stained with 10 μg/ml DAPI (4',6-diamidino-2-phenylindole) (Sigma, USA). The embryos were then mounted on glass slides and examined with a Confocal Laser-Scanning Microscope (A1R, Nikon, Japan). The steps of immunohistochemistry (IHC) were as follows. The ovaries from 8-week-old mice were fixed in 4% paraformaldehyde (Sigma) overnight at 4 °C, then dehydrated through a series of graded ethanol solutions and xylene, and embedded in paraffin (Amresco, USA). The ovaries were sectioned at a thickness of 5 μm, incubated with 1:250 anti-Myc antibody after deparaffinization at 4°C by overnight. The sections were stained with hematoxylin (Amresco) and imaged under an optical microscope (80i, Nikon, Japan).

### Western blot

Whole cell lysates from 500 embryos were lysed in 2× Laemmli buffer (0.5 M Tris HCl, pH 6.8, 0.4% SDS, 2% glycerol, 0.5% β-mercaptoethanol) and Bromophenol Blue (Bio-Rad, USA). The lysates were heated at 100 °C for 10 min, then cooled rapidly for 1 min. Proteins were separated by using SDS polyacrylamide gel electrophoresis (90 V for 30 min, 110 V 100 min,) and blotted onto nitrocellulose membranes (Biosharp, China). The membranes were blocked in TBST (TBS containing 0.1% Tween 20) containing 5% non-fat milk at room temperature 1 h, followed by overnight incubation with antibody at 4°C. After being probed with the primary antibodies, the membranes were washed in TBST, incubated with an HRP-linked secondary antibody for 1 h at 37°C, and washed three times with TBST. The bound antibodies were detected with SuperSignal West Femto Substrate Trial Kit (Thermo). The intensity of the protein bands was calculated by ImageJ software (NIH, Bethesda, USA).

### ChIP-qPCR analysis

The ChIP assays were performed according to the protocol of ultra-low-input chromatin immunoprecipitation (ULI-ChIP) 41. Samples of 2-cell embryos of si-Control and si-*Utx*-injected group were harvested for ChIP. 500 embryos were used for each reaction and three replicates were performed. 2 μg of either anti-UTX (KDM6A) or anti-IgG was used for each immunoprecipitation (IP) reaction. ChIP-qPCR were detected using a TB Green Premix Ex Taq (Takara), and the signals were detected with ABI7500 Real-Time PCR System (Thermo). Primers used for ChIP-qPCR were listed in Table S2.

### Statistical Analysis

Independent *t*-tests were performed using SPSS software version 22.0 (SPSS Inc., Chicago, IL, USA) to compare difference between two groups. A *P*-value < 0.05 was considered significant.

## Supplementary Material

Supplementary figures and tables.

## Abbreviations

MZT: maternal to zygotic transition
ZGA: zygotic genome activation
UTX: ubiquitously transcribed X chromosome tetratricopeptide repeat protein
ESCs: embryonic stem cells
ZSCAN4: zinc finger and SCAN domain containing 4
MuERVL: murine endogenous retrovirus-L
Tcstv1/3: 2-cell-stage, variable group, member 1/3
siRNA: short interfering RNA
PMSG: pregnant mare serum gonadotropin
hCG: human chorionic gonadotropin
BSA: bovine serum albumin
PBS: phosphate-buffered saline
DAPI: 4',6-diamidino-2-phenylindole
SD: standard deviation
SEM: standard error of the mean
qPCR: quantitative real-time PCR
RT-qPCR: reverse transcription-qPCR
IF: immunofluorescence
IHC: immunohistochemistry
WB: western blot
ChIP: chromatin immunoprecipitation
WT: wild-type
SCNT: somatic cell nuclear transfer
NANOG: Nanog homeobox
CDX2: caudal type homeobox 2
ICM: inner cell mass
TE: trophectoderm
