# Supplementary material for: Histone Demethylase UTX is an Essential Factor for Zygotic Genome Activation and Regulates Zscan4 Expression in Mouse Embryos
Source: Int J Biol Sci. 2019 Aug 24;15(11):2363–72. doi: 10.7150/ijbs.34635 (PMC6775313; doi:10.7150/ijbs.34635)
Supplement: Supplementary file 1 — Supplementary figures and tables. [file ijbsv15p2363s1.pdf]

Supplementary Materials

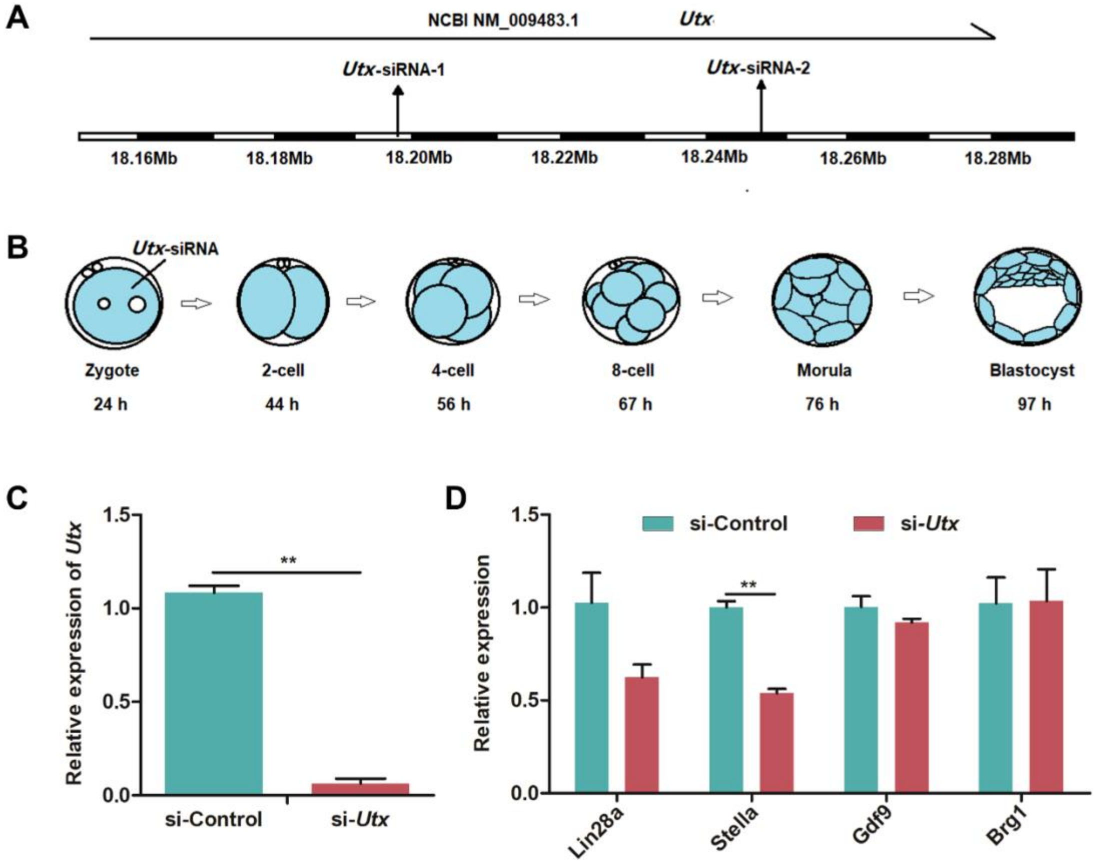

Figure S1. Functional verification of *Utx*-siRNA. (A) Schematic representation of *Utx*-siRNA. Two siRNAs were designed to target different regions of *Utx*. (B) Schematic illustration of *Utx*-siRNA injected into zygotes and *in vitro* culture. (C) qPCR results showing mRNA level of *Utx* in si-*Utx*-injected 2-cell embryos. Error bars indicate SEM. Values were normalized to *Gapdh*. $^{**}P < 0.01$ by the two-tailed Student's *t*-test. (D) qPCR results showing mRNA levels of maternal effector genes in si-*Utx*-injected 2-cell embryos. Error bars indicate SEM. All values were normalized to *Gapdh*. $^{**}P < 0.01$ by the two-tailed Student's *t*-test.

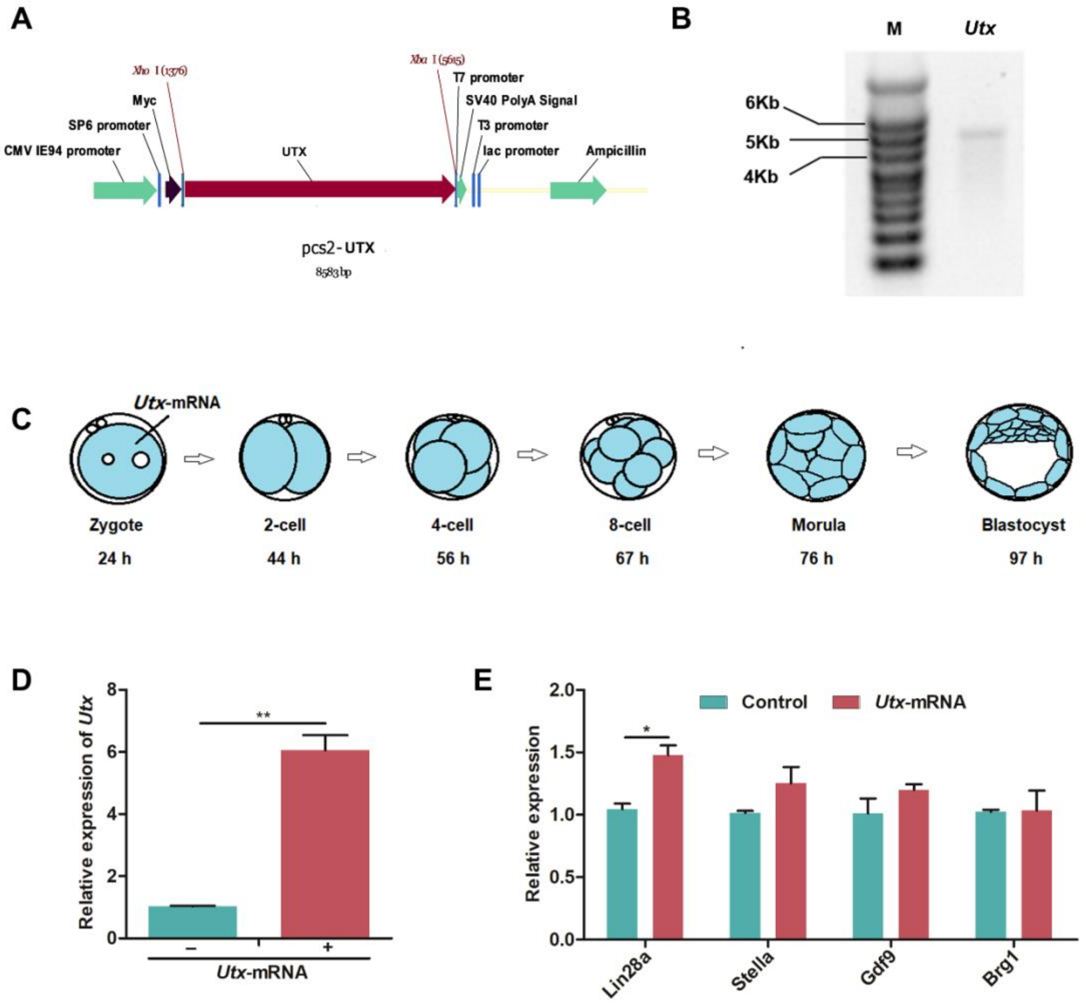

Figure S2. Functional verification of *Utx*-mRNA. (A) Schematic representation of pcs2-UTX plasmid. (B) The sketch of *in vitro* transcribed *Utx*-mRNA integrity was confirmed by electrophoresis with formaldehyde gels. M, marker. (C) Schematic illustration of *Utx*-mRNA injected into zygotes and *in vitro* culture. (D) qPCR results showing mRNA level of *Utx* in *Utx*-mRNA-injected 2-cell embryos. Error bars indicate SEM. Values were normalized to *Gapdh*. ***P* < 0.01 by the two-tailed Student's *t*-test. (E) qPCR results showing mRNA levels of maternal effector genes in *Utx*-mRNA-injected 2-cell embryos. Error bars indicate SEM. All values were normalized to *Gapdh*. **P* < 0.05 by the two-tailed Student's *t*-test.

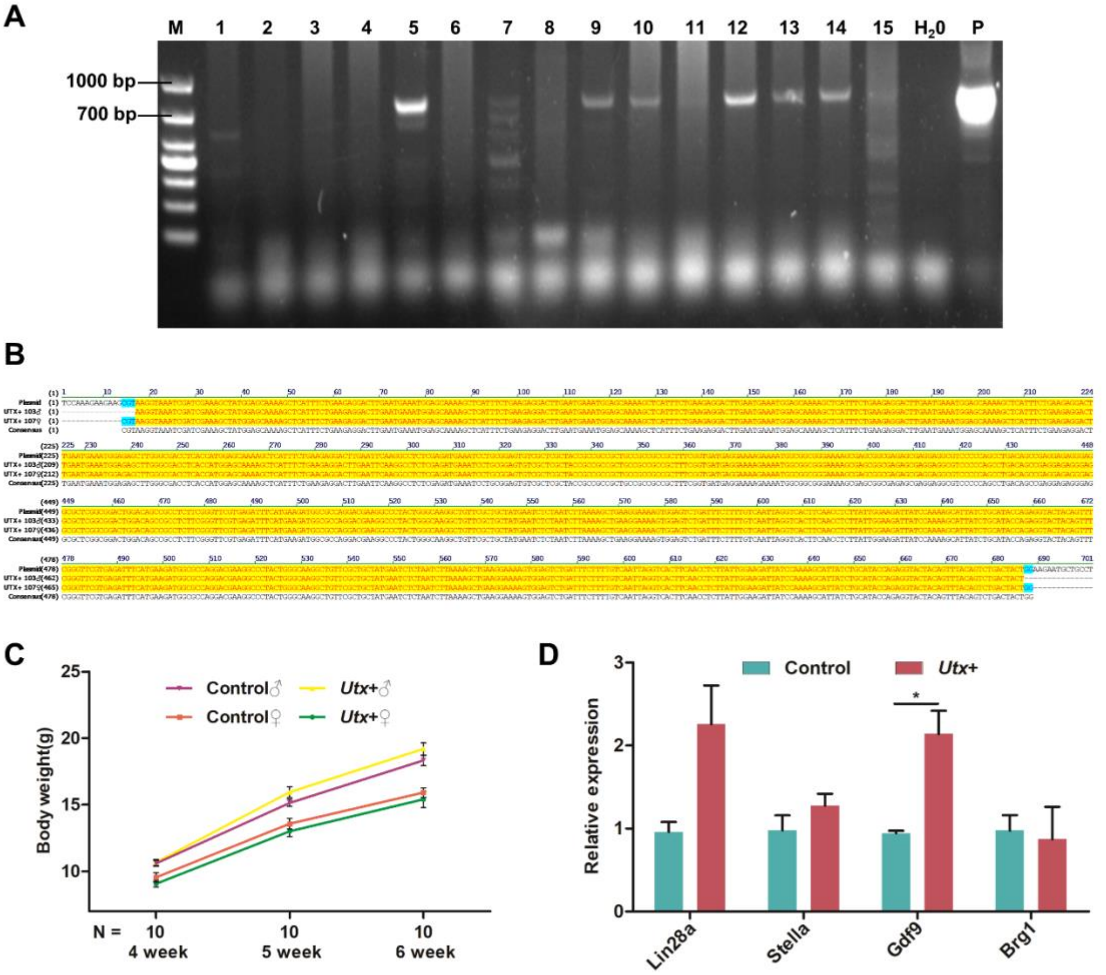

Figure S3. Identification of *Utx*+ mice. (A) PCR analysis of the *Utx*+ mice. Genomic DNA was amplified with specific primers (Table S2) detecting the *Utx*+ mice. M, marker. 1–15, PCR templates were *Utx*+ mice genomic DNA. H_2O, PCR template was H_2O. P, PCR template was pcs2-UTX plasmids. (B) Sanger sequencing alignment of *Utx*+ mice PCR products. (C) Body weight of *Utx*+ mice for 3 consecutive weeks. ♂ and ♀ indicate the male and female, respectively. N, total number of mice in each group. Error bars indicate SEM. (D) qPCR results showing mRNA levels of maternal effector genes in *Utx*+ mice 2-cell embryos. Error bars indicate SEM. All values were normalized to *Gapdh*. **P* < 0.05 by the two-tailed Student's *t*-test.

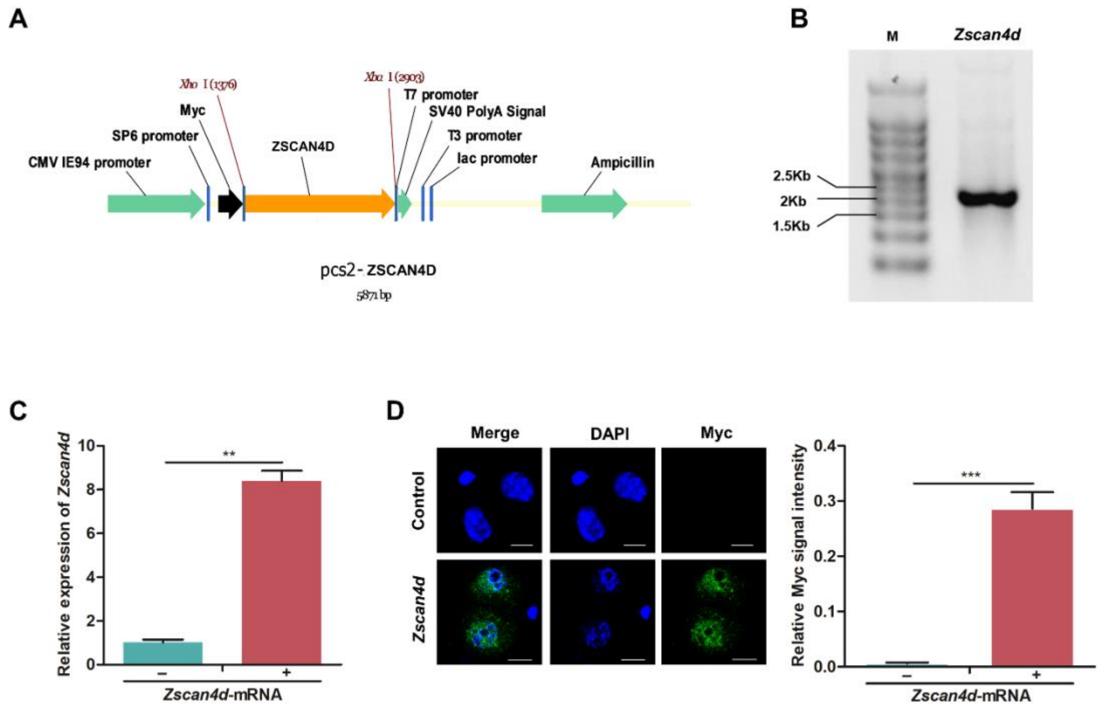

Figure S4. Functional verification of *Zscan4d* mRNA. (A) Schematic representation of pcs2-ZSCAN4D plasmid. (B) The sketch of *in vitro* transcribed *Zscan4d* mRNA integrity was confirmed by electrophoresis with formaldehyde gels. M, marker. (C) qPCR results showing mRNA levels of *Zscan4d* in *Zscan4d*-mRNA-injected 2-cell embryos. Error bars indicate SEM. Values were normalized to *Gapdh*. $^{**}P < 0.01$ by the two-tailed Student's *t*-test. (D) Immunofluorescence of Myc in *Zscan4d*-mRNA-injected embryos at the 2-cell stage (left), and quantification of Myc signal intensity (right). For the immunofluorescence images, bar graphs show the relative intensities of Myc/DAPI signal ratios. Error bars indicate SEM. Representative images from ⩾ 20 embryos analyzed using Image J; independent micromanipulations for each condition are shown. Scale bar, 20 μm. $^{***}P < 0.001$ by the two-tailed Student's *t*-test.

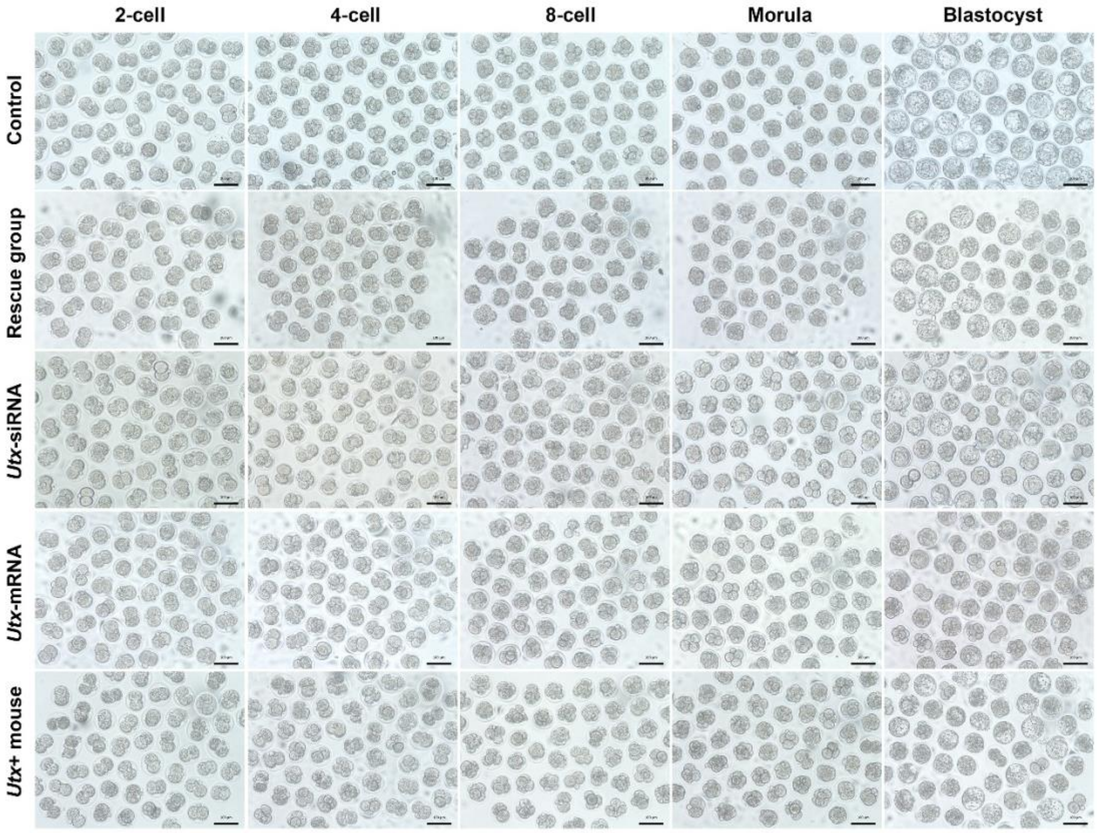

Figure S5. Bright field representative images of the embryos of the rescue (*Utx*-siRNA+*Zscan4d*-mRNA), and the control, *Utx*-siRNA, *Utx*-mRNA, and *Utx*+ mice, respectively at different culture times. Scale bar, 100 μm.

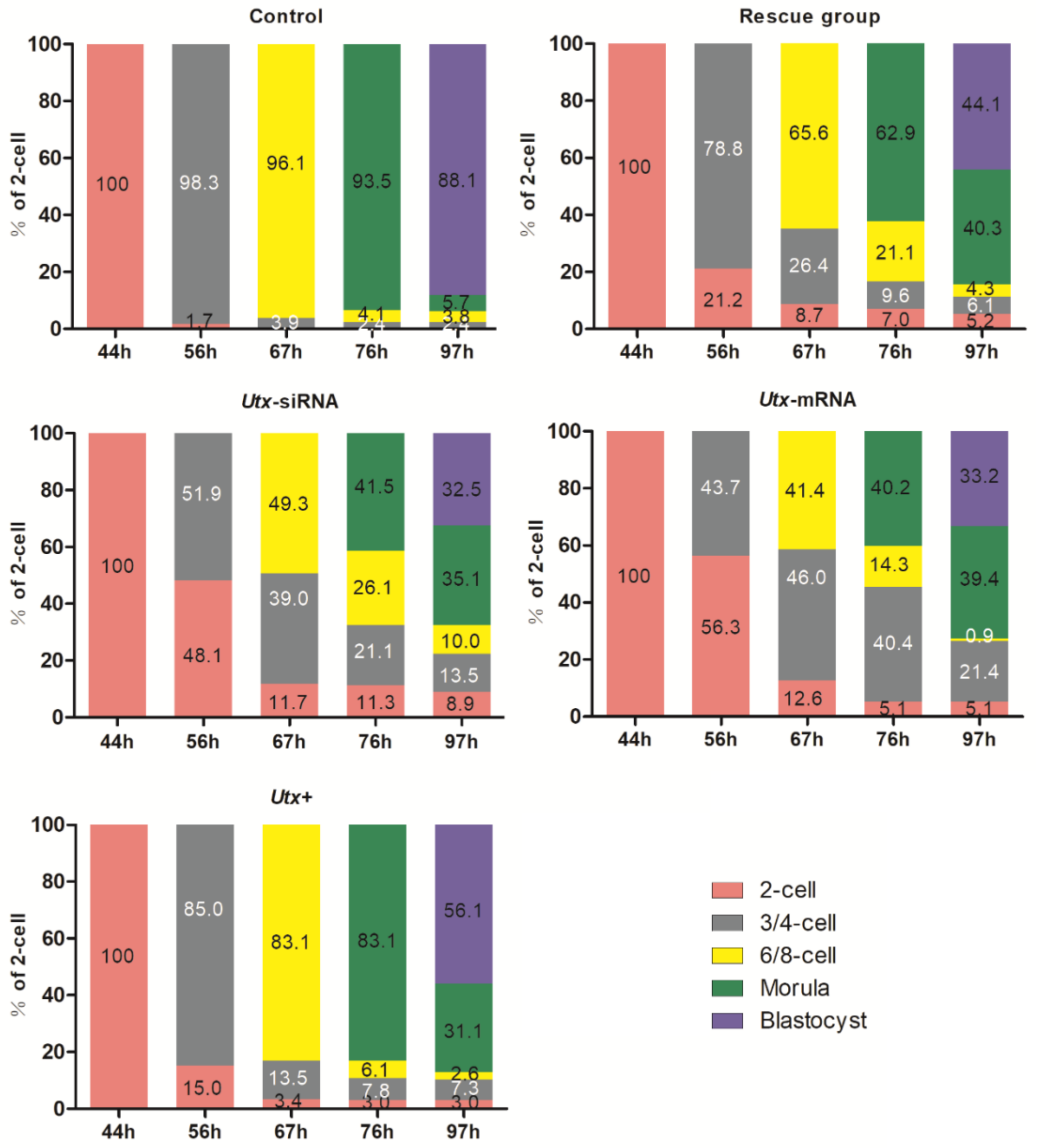

Figure S6. Development rates in the control, rescue, *Utx*-mRNA-injected, *Utx*-siRNA-injected and *Utx*+ mice groups, respectively. The efficiency was calculated based on the number of 2-cell embryos. $n \geqslant 3$.

Table S1. Developmental rate of embryos

Groups	No. of replicates	No. of 2-cell embryos	%4-cell per 2-cell±S.D.	%8-cell per 2-cell±S.D.	%morula per 2-cell±S.D.	%blastocyst per 2-cell±S.D.
Control	3	228	98.33 ± 0.7	96.06 ± 2.68	93.53 ± 2.33	88.1 ± 3.02
Rescue	3	209	78.8 ± 8.92$^{n.s.}$	65.63 ± 8.59$^{n.s.}$	62.93 ± 6.27*	44.1 ± 9.34*
Utx-mRNA injection	3	284	43.66 ± 11.59*	41.43 ± 13.24*	40.33 ± 14.24*	33.2 ± 13.59*
Utx-siRNA injection	3	278	51.93 ± 15.59*	49.3 ± 14.85*	41.53 ± 10.54*	32.5 ± 4.3**
Utx+ mice	3	176	84.99 ± 0.09*	83.1 ± 1.98$^{n.s.}$	83.1 ± 1.98$^{n.s.}$	55.98 ± 3.12**

*P < 0.05, **P < 0.01 as compared with the control group, by two-tailed Student's *t*-test. *n.s.*, not significant.

Table S2. All primers used in this study

Gene	Application	Sequence (5' - 3')
Utx	Real-time qPCR	Forward-TATTGGCCCAGGTGACTGTGAA<br>Reverse-CAAATCTCCAGGTCGCTGAATAAAC
MuERVL	Real-time qPCR	Forward-CTCTACCACTTGGACCATATGAC<br>Reverse-GAGGCTCCAAACAGCATCTCTA
Zscan4d	Real-time qPCR	Forward-TGCTTGAAGCCTCCTGTCAT<br>Reverse-GTGTTGGCCTTGTTGCAGAT
Tcstv1	Real-time qPCR	Forward-GGATCCCTGAAGGTAAATCCTC<br>Reverse-AACCATCCATCCTCAGGAAC
Tcstv3	Real-time qPCR	Forward-AGAAAGGGCTGGAACTTGTGACCT<br>Reverse-AAAGCTCTTTGAAGCCATGCCCAG
Cdc2	Real-time qPCR	Forward-GGACTACAAGAACACCTTTC<br>Reverse-CAGGAAGAGAGCCAACGGTA
eIF-1a	Real-time qPCR	Forward-TTTGGTCACTACTCAGGAGG<br>Reverse-ATCAGAAGCAACTGGGACAC
Rif1	Real-time qPCR	Forward-GCAAGGATGTTGAGACTGAGC<br>Reverse-TAGAGGCACTGGCAAGTATGTC
Rpl23	Real-time qPCR	Forward-CATGGTGATGGCCACAGTTA<br>Reverse-GACCCCTGCGTTATCTTCAA
U2afbp-rs	Real-time qPCR	Forward-TAAGCTGCAACCTGGAACCT<br>Reverse-CCTGCGTACCATCTTCCATT
Ube2a	Real-time qPCR	Forward-AATGGTTTGGAATGCGGTCA<br>Reverse-TGTTTGCTGGACTATTGGGA
Mt1a	Real-time qPCR	Forward-CACCAGATCTCGGAATGGAC<br>Reverse-AGCAGCTCTTCTTGCAGGAG
Wee1	Real-time qPCR	Forward-AGCCATCTACCGAAAGCAGA<br>Reverse-ATCTGTGAAGAGTGCCCGTT
Lin28a	Real-time qPCR	Forward-GCGAAGATCCAAAGGAGACA

		Reverse-TGTGGATCTCTTCCTCTTCC
Stella	Real-time qPCR	Forward-TGTTGTCGGTGCTGAAAGAC<br>Reverse-CACTGTCCCGTTCAAACTCA
Gdf9	Real-time qPCR	Forward-TTGGCAGTCTCTTCAGTCCA<br>Reverse-GGGAGATCTTTCCACCTCAA
Brg1	Real-time qPCR	Forward-CGGCAGAAGATTGAGAAGGA<br>Reverse-CCCAGCTTGATCTTCACCTT
Gapdh	Real-time qPCR	Forward-GTGGCAAAGTGGAGATTGTTG<br>Reverse-CTCCTGGAAGATGGTGATGG
Utx-M	Genotyping of transgenic mice	Forward-TCCAAAGAAGAAGCGTAAGGTAA<br>Reverse-AGGCAGCATTCTTCCAGTAGTCA
Zscan4d-P	ChIP-qPCR	Forward-AATCCAACCTTTCTCCCTCCAAT<br>Reverse-ACAGAGCCATACATCCACCCAAT
Zscan4d-E	ChIP-qPCR	Forward-ATCAAGAAGATAGGGCAAGAAGA<br>Reverse-ATTATGTCTAGGCATACAAGGGA
